# Modelling CD4 reagent usage across a national hierarchal network of laboratories in South Africa

**DOI:** 10.4102/ajlm.v12i1.2085

**Published:** 2023-05-15

**Authors:** Naseem Cassim, Lindi-Marie Coetzee, Deborah K. Glencross

**Affiliations:** 1 Department of Molecular Medicine and Haematology, Faculty of Health Sciences, University of the Witwatersrand, Johannesburg, South Africa; 2 National Priority Programme, National Health Laboratory Service, Johannesburg, South Africa

**Keywords:** HIV, CD4, efficiency, reagents, laboratory

## Abstract

**Background:**

The National Health Laboratory Service is mandated to deliver cost-effective and efficient diagnostic services across South Africa. Their mandate is achieved by a network of laboratories ranging from centralised national laboratories to distant rural facilities.

**Objective:**

This study aimed to establish a model of CD4 reagent utilisation as an independent measure of laboratory efficiency.

**Methods:**

The efficiency percentage was defined as finished goods (number of reportable results) over raw materials (number of reagents supplied) for 47 laboratories in nine provinces (both anonymised) for 2019. The efficiency percentage at national and provincial levels was calculated and compared to the optimal efficiency percentage derived using pre-set assumptions. Comparative laboratory analysis was conducted for the provinces with the best and worst efficiency percentages. The possible linear relationship between the efficiency percentage and call-outs, days lost, referrals, and turn-around time was assessed.

**Results:**

Data are reported for 2 806 799 CD4 tests, with an overall efficiency percentage of 84.5% (optimal of 84.98%). The efficiency percentage varied between 75.7% and 87.7% between provinces, while within the laboratory it ranged from 66.1% to 111.5%. Four laboratories reported an efficiency percentage ranging from 67.8% to 85.7%. No linear correlation was noted between the efficiency percentage, call-outs, days lost, and turn-around time performance.

**Conclusion:**

Reagent efficiency percentage distinguished laboratories into different utilisation levels irrespective of their CD4 service level. This parameter is an additional independent indicator of laboratory performance, with no relationship with any contributing factors tested, that can be implemented across pathology disciplines for monitoring reagent utilisation.

**What this study adds:**

This study provides an objective methodology to assess reagent utilisation as an independent measure of laboratory efficiency. This model could be applied to all routine pathology services.

## Introduction

The National Health Laboratory Service (NHLS) provides essential laboratory testing to the public health sector in South Africa.^1^ The organisation is mandated to provide cost-effective, reliable diagnostics to all public health facilities, irrespective of size, location, or level of complexity.^1^ A network of well-placed laboratories, ranging from highly sophisticated and centralised academic facilities to small rural, hospital-based laboratories, provide essential services^1^ for both communicable (like HIV and tuberculosis) and non-communicable diseases (cancer, diabetes, and cardiovascular diseases).^1^

Laboratory services support a vast testing repertoire, but this study focused on HIV-related diagnostics prescribed by the World Health Organization and the national treatment guidelines.^2,3^ People living with HIV are eligible for antiretroviral therapy irrespective of age, CD4 cell count, and clinical stage,^2^ and it is recommended that antiretroviral therapy is initiated within seven days if there are no clinical contraindications.^2^ Laboratory services play a key role in the baseline clinical evaluation that includes confirming HIV status, screening for tuberculosis, cryptococcal disease, renal insufficiency and determining CD4 count (to assess immune status), among others.^2^ Once patients are on antiretroviral therapy, routine HIV viral load monitoring is required, with CD4 monitoring only recommended for patients on cotrimoxazole preventive therapy.^2^

CD4 services in South Africa are offered through an integrated tiered service delivery model that aims to extend coverage, improve turn-around time (TAT), and contain programmatic costs.^4,5^ Since the implementation of the integrated tiered service delivery model, multiple remote areas with existing community laboratories that previously referred samples have been upgraded to include CD4 services, such as in De Aar, Upington, and Tshwaragano. The local CD4 testing in the newly established remote laboratories consequently reduced CD4 testing referrals, decentralising CD4 services, and caused a dramatic reduction in the TAT of CD4 test results.^6^

One way of monitoring instrument performance is through the assessment of TAT. Turn-around time assessment measures and monitors laboratory testing efficiency and result delivery to support patient care. Thus, substantial emphasis is placed on laboratory TAT, with each test having a predetermined cut-off target to ensure accurate testing and timely results. Various local studies reported a substantial improvement in TAT performance across the network of CD4 testing facilities, with continuous monitoring through a weekly TAT dashboard ensuring timely intervention where the TAT target is unmet.^7,8,9,10^

For laboratories to meet their TAT targets, they must streamline all aspects of their workflow; this includes optimising sample preparation and reagent use. Effective reagent utilisation notably saves time and money and can be used as an additional measure of laboratory performance and efficiency. Less efficient laboratories can be identified by assessing laboratory CD4 reagent utilisation, corrective practices implemented, and streamlined workflow processes introduced. Furthermore, as all CD4 testing procedures are standardised across the NHLS, including the adoption of good laboratory practice principles, practices of effective reagent utilisation can offer additional value in the quest for better service efficiencies.

‘Six Sigma’ is a quality management strategy that aims to improve the quality of processes and focuses on identifying and removing defects.^11,12^ This strategy includes ‘Lean’, a methodology used to improve the efficiency of clinical laboratory procedures.^11,12^ One of the key factors is to identify the value stream and remove wastage.^13^ In the delivery of any efficient service, lean manufacturing concepts include the flow of raw material, work in progress, and finished goods, while in the case of this study, laboratory staff, analysers, and information elucidate efficiency.^13,14^ In a pathology setting, the flow of raw materials relates to the supply of test reagents and associated consumables required to perform a specific test. Finished goods in a laboratory environment refer to the verified result delivered to the healthcare worker or originator of the test request. To produce a result, the laboratory requires trained staff to conduct testing on appropriate analysers before test results are verified on the laboratory information system (LIS) for reporting.

The study assessed laboratories’ optimal use of CD4 test reagents to identify laboratories that need to minimise wastage by improving and refining their workflow. The laboratories utilised national, standardised analyser platforms and protocols (standard operating procedures) and a national LIS. This study aimed to model CD4 reagent use across a national network of laboratories, using data for the 2019 calendar year in South Africa. A secondary objective was to review national and provincial efficiency of reagent use and reagent usage at the individual laboratory level. In laboratories where efficiency was suboptimal, a further objective was to assess whether there was any correlation between their reagent efficiency percentage and other service efficiency indicators. These service indicators included the number of service call-outs during instrument failure, days lost (as a consequence of instrument downtime), delays due to referrals (where samples are sent to sister laboratories for testing during instrument downtime), and TAT performance of the affected laboratory.

## Methods

### Ethical considerations

Ethical clearance for this study was obtained from the University of the Witwatersrand (M220163). Only aggregate secondary laboratory data were used; our study did not require the use of any patient identifiers. All specific province and individual laboratory identifiers were removed, and sites were anonymised for this study. The Human Research Ethics Committee did not require patient consent.

### Study design

The cross-sectional study design assessed the CD4 reagent utilisation of 47 NHLS laboratories in South Africa for the 2019 calendar year. CD4 testing was performed on the Beckman Coulter FC500 MPL/CellMek and Aquios CL cytometers (Beckman Coulter, Miami, Florida, United States) during the studied period. Beckman Coulter supplied all the CD4 reagents for both cytometers per the national tender agreement.^15,16^ Irrespective of the instrument used, all laboratories utilised the same CD4 PanLeucoGating reagents and national standard operating procedures.^15^

### Optimal efficiency percentage calculation

The optimal efficiency percentage per 100 tests was calculated using over 18 years of CD4 testing data (from 2004) of the National Priority Programme.^17,18^ To determine the optimal efficiency percentage, we assumed an 8-h working day and a 5-day testing week and included error rates, the number of controls used (which count as one test per control tested), days lost (due to instrument downtime), and other indicators (such as electricity outages).

### Data extraction

Beckman Coulter (Miami, Florida, United States) provided aggregate data on raw materials (test reagent kits) delivered to each laboratory for 2019, and the number of tests provided from each delivery was estimated by multiplying the number of kits delivered by 300 (the number of tests per kit). The variables for this extract included: (1) reagent item number (the company’s reagent kit product identifier), (2) ‘ship-to location’ used for supply chain management (which indicates the laboratory that received the kits), and (3) number of kits delivered. In addition, Beckman Coulter also provided the number of service call-outs for each laboratory during 2019.

In this study, finished goods were defined as the number of CD4 results authorised by qualified technical personnel on the LIS (for this study, this process is termed ‘review’). The reported test volumes, identified per laboratory, were extracted by the Corporate Data Warehouse. The specimen-level CD4 variables reported were: (1) episode number, (2) testing laboratory, (3) source laboratory, (4) province, (5) review date, (6) TAT (in hours), and (7) referral status (referred or not referred). The source laboratory may not routinely offer CD4 services and would have to refer samples to a testing facility.

### Data preparation

The Beckman Coulter ‘ship-to location’ was matched to the respective equivalent-identified Corporate Data Warehouse testing laboratory, for example ‘Bongani Hospital’ with ‘Welkom’. The raw and finished materials were used to calculate the efficiency percentage (Finished Goods/Raw Materials). The analysis was conducted using Microsoft Excel (Microsoft Corp., Redmond, Washington, United States). All provinces and laboratories have been anonymised, for example Laboratory 1 (P3), to ensure confidentiality.

### Data analysis

The following indicators were reported for each laboratory: (1) efficiency percentage, (2) number of call-outs (calls logged with the supplier helpdesk), (3) days lost, (4) sample referral percentage, (5) samples meeting TAT cut-off percentage, and (6) capacity utilisation. Microsoft Excel was used for the capacity utilisation by calculating the throughput, for example 120 samples per 8-h shift for the Aquois flow cytometer, and the test volumes performed for a defined period.^15^ For this analysis, we divided the annual test volumes by annual capacity, assuming hours worked and the number and type of flow cytometer platform used per laboratory. For each laboratory, the total number of call-outs logged with the Beckman Coulter call centre was reported. Call-outs were categorised as field mentoring, field service visits, or modification type 2 (software or hardware update) reports. In consultation with Beckman Coulter, call-outs related to non-essential support from the supplier, including activities such as courtesy visits or initial instrument installation, were not deemed to impact service delivery and efficiency and were disregarded.

Daily laboratory test volumes were analysed to determine the total number of days lost. A lost day was defined as a weekday (Tuesday to Friday) where fewer than 15 tests were performed. Test volumes over weekends, public holidays and Mondays were excluded from this analysis.^19^ Monday test volumes were excluded because, historically, there are limited HIV services on this day, resulting in low test volumes.^19^ The daily test volumes were coded using this threshold as 1 or 0 to determine the number of lost days.

The laboratory data were used to determine the percentage of samples that were referred (where the source and testing laboratory were different in the LIS). A referral was defined as a sample originating from a different laboratory from where it was tested. Referrals involve the transportation of samples with delays that could affect sample stability. All references to referrals in the manuscript relate to the percentage of referred samples.

A key laboratory efficiency report is the TAT performance for various tests.^20^ Turn-around time assesses laboratory service delivery speed, reliability, and efficiency.^20^ The NHLS agrees to an annual performance plan with the National Department of Health, which includes key outcomes and outputs.^20^ The optimal national CD4 reporting TAT target for 2019 was 90% of samples tested within 40 h. This TAT allows timely baseline laboratory investigations to ensure antiretroviral therapy initiation within seven days.^2^ All laboratories within the national CD4 network are required to meet the organisation’s CD4 TAT targets, which are monitored by measuring the percentage of samples that any given laboratory reports within the 40-h window. For this study, this measurement was also used and is shown as the percentage of samples that met the TAT cut-off of 40 h, referred to as TAT performance, for comparison to other efficiency parameters reported.

A Microsoft Excel bubble chart was used to report the reagent efficiency percentage, the percentage of samples that met the organisational TAT target, and annual raw material cost (in United States dollars). Laboratory performance was then categorised as a scatter plot reporting the reagent use efficiency by TAT performance.

To assess any linear relationship to the percentage reagent efficiency and call-outs, days lost, referrals, and TAT performance scatter plots were generated for all laboratories using Stata^®^ SE (StataCorp LLC, College Station, Texas, United States). Pairwise correlation coefficients between these variables were also determined.

For the top and bottom performing provinces (with the lowest and highest percentage reagent efficiency), the following parameters were tabled for each testing laboratory within these provinces: (1) efficiency percentage, (2) number of call-outs, (3) days lost, (4) percentage of referrals, and (5) percentage of samples that met the TAT cut-off. An analysis of the instrument capacity utilisation for these laboratories was also conducted.

## Results

We report the data for 2 806 799 CD4 tests performed in 2019 across 47 testing laboratories. There are nine provinces with 3–10 testing laboratories per province.

### Optimal efficiency percentage

We assumed an error rate of 8%, four controls per day, 4 days lost per year and 3% for other interruptions for an optimal efficiency percentage. The days lost were calculated using 50 weeks and five working days per week. Two weeks were excluded to account for annual public holidays.^21^ Thus, the calculated optimal efficiency percentage was 84.98% (Table 1), and indicates that for a typical kit of 300 tests, each laboratory, in an optimal setting, should be able to produce 254 reportable CD4 results. The remaining 15% (46 tests per kit) accounts for other aspects such as daily controls, error rates, and other testing interruptions (i.e. power outages or instrument downtime).

**TABLE 1:** Assumptions and calculation of the optimal CD4 reagent efficiency percentage, South Africa, 2019.

Assumption	Assumption applied	Samples affected	Net samples	Efficiency percentage
Batch of 100 samples	-	100	100	100
Error rate	8%	8	92	92
Controls	4 per day	4	88	88
Days lost†	4 per year	0.016	87.984	87.98
Other (power outage, etc.)	3%	3	84.984	84.98

† , Based on 50 weeks and five working days considering public holidays. There are 12 public holidays per year in South Africa.^21^

### National and provincial analysis

**FIGURE 1:**
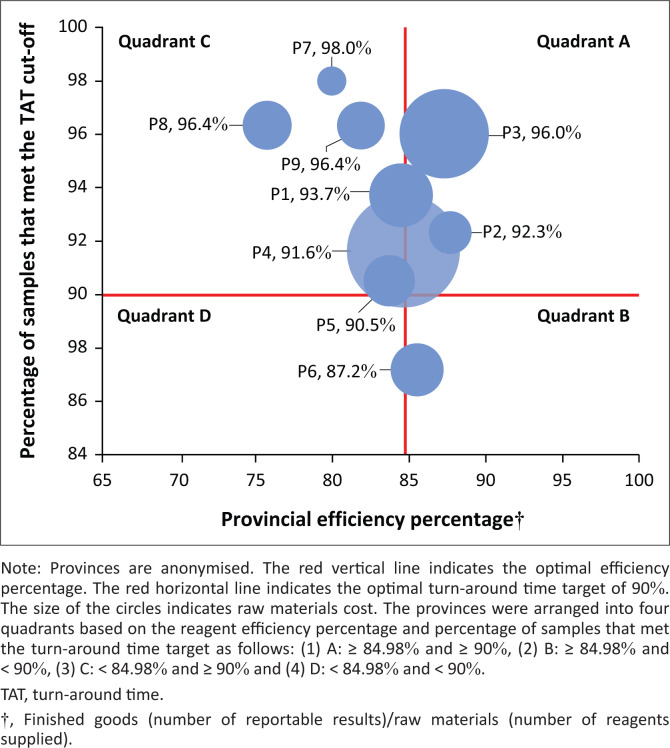
Bubble chart assessing provincial CD4 reagent utilisation of nine provinces in South Africa for 2019. The efficiency percentage, percentage of samples that met the turn-around time target, and raw materials cost (United States dollars) are reported on the *x*-axis, *y*-axis, and as bubble size.

A national efficiency percentage of 84.5% was reported across all CD4 laboratories during 2019 (data not shown). The provincial analysis revealed that 53.7% of all finished materials (CD4 results) were generated by two provinces (data not shown). The provincial efficiency percentage ranged from 75.7% (P8) to 87.7% (P2) (Figure 1). Only two provinces reported an efficiency percentage of ≤ 80% (P8: 75.7% and P7: 79.9%). The percentage of samples that met the TAT target ranged from 87.2% (P6) to 98.0% (P7).

### Laboratory reagent efficiency

**FIGURE 2:**
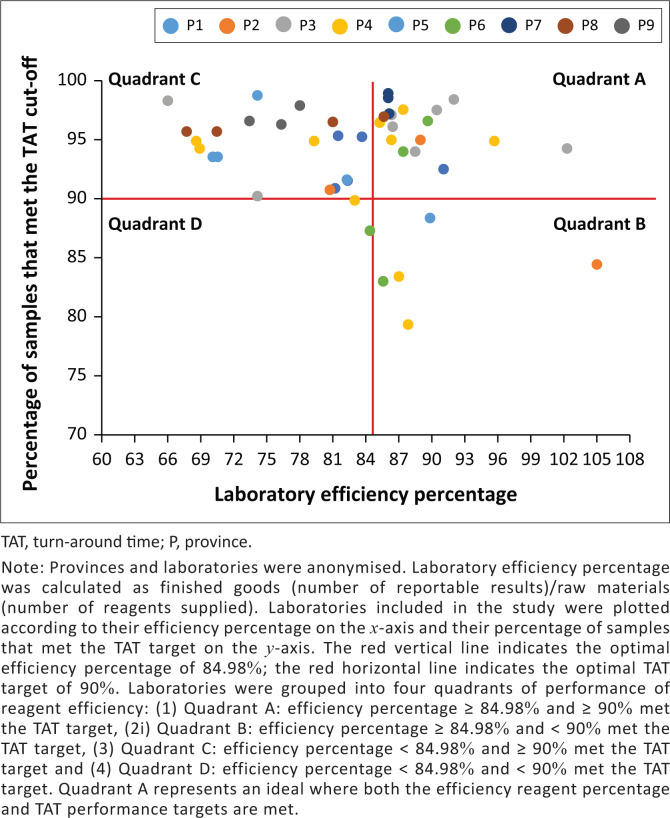
CD4 reagent utilisation of 47 laboratories across nine provinces in South Africa for 2019.

The laboratory efficiency percentages ranged from 66.1% (Laboratory 13 [P3]) to 104.5% (Laboratory 22 [P2]) (Figure 2). Other indicators of laboratory efficiency also varied; the percentage of samples that met the stipulated organisational CD4 TAT cut-off varied from 79.5% (Laboratory 19 [P4]) to 99.0% (Laboratory 3 [P7]). The days lost to testing ranged from 1 day (Laboratory 1 [P3]) to 52 days (Laboratory 22 [P2]) (Figure 2). Overall, 21/47 laboratories met the optimal efficiency percentage target (44.7%) compared to 40/47 for TAT performance (85.1%). Quadrants A and C met the national stipulated TAT target (> 90% of reported CD4 results with 40 h). Quadrant A (comprising 36.2% or 17/47 laboratories) showed efficient reagent use (≥ 84.98%), while Quadrant C (comprising the majority of laboratories, i.e. 53.2% or 25/47 laboratories) showed less efficient reagent usage (< 84.98%). Quadrants B and D did not meet the stipulated TAT; however, Quadrant B (8.5% or 4/47 laboratories) showed efficient use of reagents ≥ 84.98%, while Quadrant D (2.1% or 1/47 laboratories) reported < 84.98% reagent efficiency.

### Correlation of reagent efficiency percentage to other factors

**FIGURE 3:**
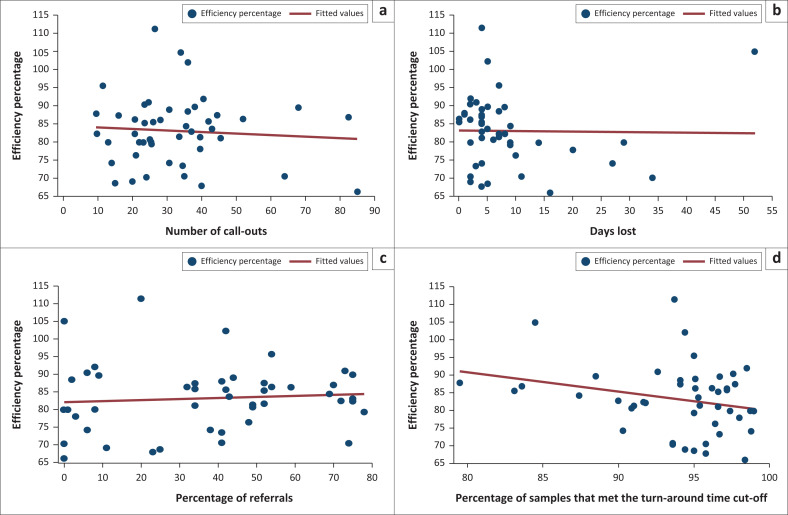
Correlation of CD4 reagent use efficiency of 47 laboratories in South Africa for 2019. Efficiency is plotted with (a) the number of call-outs, (b) the number of days lost, (c) the percentage of referrals, and (d) turn-around time performance.

The scatter plot analysis for all laboratories did not reveal a linear relationship between efficient reagent use and call-outs, days lost, referrals, and TAT performance (Figure 3). A pairwise correlation coefficient of −1 or +1 would indicate a perfect linear relationship^15^; however, values of −0.0770, −0.0151, 0.0853 and −0.2596 were reported for these test parameters, confirming very weak correlation (values very close to 0).

### Detailed analysis of individual laboratory efficiency for the top and bottom provinces

The laboratories in the bottom province (P8) had an efficiency percentage ranging from 67.8% to 85.7% (Table 2). Only one laboratory in this province met the optimal efficiency percentage (Laboratory 20). The number of call-outs for these laboratories varied from 42 to 64; however, days lost to service delivery in these same four sites ranged between 2 and 4 days. The percentage of referred samples tested in these sites also varied from 23.4% to 41.0%. Conversely, all four laboratories had TAT values ranging from 95.8% to 97.2%. The instrument capacity utilisation was below 85% for all testing sites (Table 2).

**TABLE 2:** Comparison of the laboratories with highest and lowest CD4 reagent use efficiency, South Africa, 2019.

Category	Laboratory and (Province) alias	Efficiency percentage	Number of call-outs	Days lost	Percentage referrals	Percentage within turn-around time cut-off	Percentage instrument capacity utilisation
Worst province	Lab 32 (P8)	67.8	40	4	23.4	95.8	58.8
	Lab 12 (P8)	70.5	64	2	41.0	95.8	66.2
	Lab 39 (P8)	81.0	46	4	34.2	96.6	67.8
	Lab 20 (P8)	85.7	42	4	34.3	97.2	84.7
Best province	Lab 46 (P2)	80.7	25	6	49.2	90.9	52.9
	Lab 26 (P2)	89.0	31	4	44.0	95.1	57.9
	Lab 22 (P2)	104.9	34	52	0.0	84.5	61.9

Note: The number of call-outs were logged with the supplier, days lost (< 15 tests performed).

In the best-performing province (P2), the reagent use efficiency percentage ranged from 80.7% to 104.9% across three laboratories. A range of 25–34 call-outs and 4–52 days lost was reported. Only Laboratory 22 reported a ≥ 90% TAT performance. Instrument capacity utilisation was under 62% for this province.

## Discussion

The work investigated how effectively CD4 laboratories used reagents to deliver CD4 services in South Africa. The outcomes revealed that the national CD4 network reported a CD4 efficiency percentage of 84.5%, ranging from 75.7% to 87.7% at the provincial level. Individual laboratory efficiency percentage went as low as 66.1%.

The averaged national performance masked less efficient reagent use at provincial and laboratory levels, evident in the variability of efficiency percentages reported for provinces and laboratories. Several laboratories had efficiency percentages below 18.9% of the calculated optimal reagent use percentage. Thus, it is essential to assess efficiency at the decentralised level to identify the less efficient performers for appropriate corrective action and intervention. Similar masking of laboratory performance was reported for TAT in the CD4 testing network.^10^

One interesting observation was a laboratory with an efficiency reagent use of over 100%. This aberration indicates that some reagents from the previous year may have been used in 2019 (for example, careful provision for contingency and reagents for the 2018 holiday period, which was then used in 2019). Diligent stock control will assist in standardising contingency reagent stocks held in laboratories.

Only 21 sites met the calculated optimal reagent efficiency percentage, indicating that perhaps the optimal efficiency percentage was too optimistic and failed to factor in laboratory testing volume differences. For example, a site with much higher CD4 processing volumes must perform relatively more control tests each day than a site with lower test volumes or that work only an 8-h shift versus two or three 8-h shifts. The detailed analysis for the top and bottom provinces also suggests that in addition to the standardised test platform, the workflow and stock management procedures need to be standardised across laboratories to ensure best practices.

Other contributing variables that could have affected the laboratory efficiency percentage include varying staff capacity (staff numbers) and expertise. These aspects are especially difficult to assess in smaller, community-type laboratories that typically employ multi-tasking rotations across pathology disciplines to deliver essential pathology services.^4,6,7^

Instrument log data files were unavailable at the time of this study. It was, therefore, impossible to assess the number of repeated tests run, the frequency of controls performed (especially as a proportion of the total volume of tests performed), and the number of invalid runs, all of which could contribute to the lower percentage efficiency, especially in smaller CD4 volume laboratories. This study, however, highlights the value of instrument log data files and the need to collate this information on an organisation’s central data server to facilitate near real-time analysis, including reagent utilisation. Other information, including days lost, duplicated testing, and the number of controls used, could also be logged and centrally monitored to identify more systemic problems in laboratories. Here, instrument-collected data would be invaluable to identify practices employed in the better-performing laboratories that could be standardised across all testing sites. Such an approach would make it easier to introduce corrective interventions and pair provinces with a poor efficiency percentage to their better-performing sister sites. Ultimately all efficiency parameters mentioned in this article should ideally be monitored at a centralised level by a national overseeing, harmonising body that uses instrument logs and other relevant data to report on national service efficiency and performance.^4,6,7^

A comprehensive monitoring and evaluation plan should be developed that stipulates the indicators to be reported. The development of appropriate dashboards reporting reagent utilisation efficiency is needed to monitor performance for continual improvement of the national laboratory system. Furthermore, this reagent efficiency model could be extended to other tests across a national laboratory network to improve reagent efficiency across all pathology disciplines.^8,9^ To accomplish this, a national LIS is key to generating the data for real-time monitoring. Well-narrated data provide far more granularity than aggregate data systems^22^ and ensure that data are continually visible from the national to the laboratory level for all tests. In our context, all laboratory data is stored in a data warehouse environment with massive server capacity for all tests in the national network.^22^

Further investigation is required to understand how reagents are used in CD4 laboratories with higher and lower volumes to improve overall reagent usage. Interestingly, the lack of linear correlation between reagent usage and other efficiency parameters, including call-outs, days lost, referrals, and TAT performance, could indicate more systemic laboratory problems. Although beyond the scope of this study, detailed on-site inspection at each laboratory, with full audits of procedures and practices, could address some of these questions.

### Limitations

A limitation of this study was the absence of instrument log file data. Unfortunately, these data are stored on a local instrument-linked computer. In addition, the optimal efficiency percentage calculated was based on averages of laboratories studied with the assumptions stated in the methods section; outlines suggest this calculated percentage may have been too optimistic and requires further workflow studies.

### Conclusion

We reported a model of reagent efficiency across a pathology network with various service tiers. The work emphasises the importance of review at all hierarchical tiers of service, in this instance at the provincial or individual laboratory level, to detect masked poorer levels of efficiency and areas for corrective action. There was no correlation between the reagent use efficiency with days lost, call-outs, referrals, and TAT performance, suggesting that although reagent usage and instrument function are both aspects that contribute to overall laboratory efficiency, these are separate issues that need to be monitored individually. Underlying factors contributing to reagent wastage should be investigated to inform waste awareness strategies and standardised procedures to minimise missed diagnostic opportunities and cost implications. Real-time monitoring of reagent use through instrument log data files can save costs, which is especially relevant in an expenditure-constrained setting.
